# Galectin-1 inhibits PDGF-BB-induced proliferation and migration of airway smooth muscle cells through the inactivation of PI3K/Akt signaling pathway

**DOI:** 10.1042/BSR20193899

**Published:** 2020-06-15

**Authors:** Xinfeng Pang, Jing Qiao

**Affiliations:** 1 Medical Laboratory Center, Jiaozuo Women’s and Children’s Hospital, Jiaozuo 454000, Henan, P.R. China; 2 Pediatric Respiratory Medicine, Jiaozuo Women’s and Children’s Hospital, Jiaozuo 454000, Henan, P.R. China

**Keywords:** airway smooth muscle cells (ASMCs), Childhood asthma, galectin-1 (Gal-1), phenotype switching, PI3K/Akt signaling pathway

## Abstract

Childhood asthma is one of the most common chronic childhood diseases. Platelet-derived growth factor BB (PDGF-BB) induced airway smooth muscle cell (ASMC) proliferation and migration are involved in the pathogenesis of asthma. Galectin-1 (Gal-1) is a glycan-binding protein that has been found to be involved in the progression of asthma. However, the mechanism remains unclear. In the current study, we aimed to evaluate the role of Gal-1 in regulating the phenotype switching of ASMCs, which is an important mechanism in the pathogenesis of asthma. Our results showed that Gal-1 was markedly down-regulated in the samples from asthma patients. *In vitro* study also proved that Gal-1 expression was decreased in PDGF-BB-stimulated ASMCs. In addition, Gal-1 overexpression significantly inhibited PDGF-BB-induced ASMCs proliferation and migration, while Gal-1 knockdown exhibits opposite effects of Gal-1 overexpression. The PDGF-BB-caused reductions in expressions of α-smooth muscle actin (α-SMA), specific muscle myosin heavy chain (SM-MHC), and calponin were elevated by Gal-1 overexpression, but were deteriorated by Gal-1 knockdown in ASMCs. Furthermore, overexpression of Gal-1 inhibited PDGF-BB-stimulated PI3K/Akt activation in ASMCs. Notably, treatment with IGF-1, an activator of PI3K, reversed the effects of Gal-1 on ASMCs proliferation, migration, and phenotype switching. In conclusion, these findings showed that Gal-1 exerted inhibitory effects on PDGF-BB-stimulated proliferation, migration, and phenotype switching of ASMCs via inhibiting the PI3K/Akt signaling pathway. Thus, Gal-1 might be a promising target for the treatment of asthma.

## Introduction

Childhood asthma is one of the most common chronic childhood diseases with high mortality and morbidity in the world [1,2]. It may cause frequently emergency department visits, hospitalizations and as well as lower quality of life, thus childhood asthma places a large burden on children and their families, even on society [3]. The current therapy with inhaled corticosteroids may cause multiple side effects, such as hormone dependence or resistance, decreased bone mineral density, and inhibition of growth in children [4,5]. Thus, new therapeutic approach should be developed to overcome these negative consequences.

Increasing researchers have focused on revealing the asthma pathogenesis, and thereby provide evidence for developing new therapies [6,7]. It has been demonstrated that asthma is a long-term inflammatory respiratory disease that may be caused by a combination of environmental and genetic factors. Airway smooth muscle cells (ASMCs) are multifunctional cells that play potential roles in the pathogenesis of asthma [8]. ASMCs hyperplasia has been regarded as the main mechanism of airway smooth muscle thickening during the progression of asthma. Emerging evidence shows that the phenotypic plasticity of ASMCs turn into a proliferative and synthetic phenotype is responsible for cellular hyperplasia [9]. Platelet-derived growth factor BB (PDGF-BB) has been reported to initiate a multitude of biological effects that contribute to ASMC proliferation and migration [10]. Hence, inhibiting the changes of ASMCs may prevent the development of asthma.

Galectin-1 (Gal-1) is a glycan-binding protein that has ability to bind to residues of complex N- and O-glycans on cell-surface glycoconjugates [11]. Gal-1 is widely distributed in many types of cells and is present in both inside cells including nucleus, cytoplasm, and inner surface of plasma membrane, as well as in outside cells such as outer surface of cell membrane and extracellular matrix [11]. Previous studies have proven that Gal-1 plays a profound role in resolving acute and chronic inflammation through regulating multiple processes, such as immune cell adhesion, differentiation, migration, proliferation, activation, apoptosis, and signaling pathways [12,13]. Interestingly, Gal-1 was reported to be involved in the progression of asthma [14–16]. However, the effects of Gal-1 on ASMCs are still unknown. In the present study, we explored the role of Gal-1 in proliferation, migration, and phenotypic plasticity of ASMCs in response to PDGF-BB stimulation.

## Materials and methods

### Sputum samples collection

A total of 24 cases of children asthma patients and 18 cases of respiratory nonasthmatic patients were obtained from the Pediatric Respiratory Medicine, Jiaozuo Women’s and Children’s Hospital (Jiaozuo, China). All subjects were diagnosed and confirmed by visit a pulmonologist. Sputum at the time of enrollment in these subjects was induced according to standard methods. The collected sputum was mixed with an equal volume of 0.1% dithiotreitol, shook for 15 min at room temperature, filtered through a 0.42 μm Millipore filter, and centrifuged at 1500×***g*** for 10 min. After removing the supernatant, the pellets were separated and stored at −80°C until further analysis. The sputum samples were used for the detection of Gal-1 level, and the usage of these samples was approved by the Institutional Review Board at Jiaozuo Women’s and Children’s Hospital. Written informed consent was obtained from each participant’s parents. The characteristics of asthmatic patients and healthy controls are shown in Table 1.

**Table 1:** The characteristics of asthmatic patients and healthy controls

Characteristics	Asthmatic patients (*n* = 24)	Healthy controls (*n* = 18)	*P*
Age	8.24±2.13	8.56±2.39	0.92
Gender (male/female)	11/13	7/11	0.89
FEV1 (%)	63.17±8.73	93.18±4.26	<0.01
Serum total IgE (IU/ml)	152.72±2.13	21.39±3.45	<0.001

FEV1, forced expiratory volume in 1 s.

### Cell culture

Human ASMCs obtained from American Type Culture Collection (ATCC, Manassas, VA, U.S.A.) were cultured in Dulbecco’s modified Eagles medium (DMEM; Invitrogen, Carlsbad, CA, U.S.A.). The DMEM was supplemented with 10% fetal bovine serum (FBS; Hyclone, Logan, UT, U.S.A.), 20 units/l penicillin, and 20 μg/ml streptomycin (Gibco, Rockville, MD, U.S.A.). ASMCs were maintained in a humidified atmosphere with 5% CO_2_ at 37°C. For the PDGF-BB group, ASMCs were stimulated with PDGF-BB (10 ng/ml; Sigma, St. Louis, MO, U.S.A.) for 24 h.

### Quantitative RT-PCR (qRT-PCR)

Total RNA was extracted from sputum samples and ASMCs using the RNeasy Mini kit (Qiagen, Valencia, CA, U.S.A.) and then reverse transcribed to single-strand cDNA using a Reverse Transcription System (Promega, Madison, WI, U.S.A.). Real-time PCR was then performed using SYBR Premix Ex TaqTM (Takara, Kyoto, Japan). The amplification was conducted on an ABI Prism 7500 instrument (Applied Biosystems, Foster City, CA, U.S.A.) with the following thermal profile: 95°C for 5 min; followed by 35 cycles, 94°C for 1 min, 62°C for 1 min, 72°C for 1 min; and final extension cycle at 72°C for 10 min. The specific primers for Gal-1 and β-actin were synthetized by Sangon Biotech (Shanghai, China): Gal-1 sense, 5′-AAC CTG GAG AGT GCC TTC GA-3′ and anti-sense 5′-GTA GTT GAT GGC CTC CAG GT-3′; β-actin sense, 5′-TTA GTT GCG TTA CAC CCT TTC-3′ and anti-sense 5′-ACC TTC ACC GTT CCA GTT T-3′. The relative gene expression of Gal-1 was determined by the method of $2^{-\text{ΔΔ}C_{\text{t}}}$.

### Western blot

ASMCs were collected and lysed with RIPA lysis buffer (Thermo Fisher Scientific, Waltham, MA, U.S.A.). The homogenates were centrifuged at 4000×***g*** for 10 min at 4°C, and then the supernatants were collected. Equal amounts of protein (50 μg/lane) were subjected to 12% SDS-PAGE, and electrotransferred onto polyvinylidene fluoride (PVDF) membranes (Millipore, Boston, MA, U.S.A.). Subsequently, the membranes were blocked with 5% non-fat milk in TBST (pH of 7.5, 10 mM Tris–HCl, 150 mM NaCl, and 0.05% Tween-20) for 1 h at room temperature. After that, the membranes were incubated with primary antibodies (diluted with TBST) against Gal-1, matrix metalloproteinase (MMP)-2, MMP-9, α-smooth muscle actin (α-SMA), specific muscle myosin heavy chain (SM-MHC), calponin, p-PI3K, PI3K, p-Akt, Akt, or β-actin (Santa Cruz Biotechnology, Santa Cruz, CA, U.S.A.) at 4°C overnight. Following washing with TBST buffer for three times, the membranes were added with HRP-labeled conjugated goat anti-rabbit IgG at room temperature for 1 h. Finally, the specific immunoreactive protein bands were developed using an enhanced chemiluminescence (ECL) detection system (Thermo). The absorbance values of the target proteins were performed through Gel-Pro Analyzer version 4.0 software (Media Cybernetics, Silver Spring, MD, U.S.A.).

### Construction of pcDNA3.1-Gal-1 vector and cell transfection

The cDNA of Gal-1 gene (*LGALS1*) was introduced into pcDNA3.1 vector and the constructs were confirmed by gene sequencing. An empty pCDNA3.1 vector was used as a control. ASMCs were seeded into a six-well cell culture plate and incubated for 24 h, followed by the transfection with pcDNA3.1-Gal-1 vector or pCDNA3.1 vector using Lipofectamine 2000 (Invitrogen).

### SiRNA targeting Gal-1 and cell transfection

Specific siRNA targeting Gal-1 (si-Gal-1) and a negative control siRNA (si-control) were obtained from Invitrogen. According to the manufacturer’s instructions, si-Gal-1 or si-control was transfected into ASMCs using Lipofectamine 2000 (Invitrogen). After 48 h post transfection, cells were collected for RT-PCR and Western blot analysis.

### Cell proliferation assay

Cell proliferation of ASMCs was evaluated using the Cell Counting Kit-8 (CCK-8) reagent (Dojindo, Kumamoto, Japan). Briefly, ASMCs were seeded in 96-well plates at 1 × 10^4^ cells/well and incubated for 24 h at 37°C. Afterwards, the supernatant was removed, and the cells were added with CCK-8 reagent for 1.5 h at 37°C. The spectrometric absorbance at 450 nm was measured with a Benchmark microplate reader (Bio-Rad, Hercules, CA, U.S.A.).

### Cell migration assay

Transwell assay was performed to evaluate cell migration using transwell chambers (Corning Incorporated, Corning, NY, U.S.A.). In brief, ASMCs (5 × 10^4^ cells/well) were resuspended in 100 μl serum-free DMEM and serum-starved overnight. And then the cells were added into the upper chamber, while 600 μl DMEM containing 10% FBS was added to the lower chamber. After incubation for 24 h, non-migrated cells remained on the upper side of the membranes were removed using a cotton bud, whereas the cells migrated to the lower side of the membranes were fixed with 95% methanol and stained with 0.1% crystal violet. Five random optical fields were imaged and the number of migrated cells from was counted under a microscope.

### Data analysis

All experiments were carried out in triplicates and then analyzed using SPSS v.16.0 (SPSS Inc., Chicago, IL, U.S.A.). The data were expressed as mean ± S.D. differences between two groups/among multiple groups were evaluated using the Student’s *t*-test or one-way ANOVA. The *P* values less than 0.05 were considered significant.

## Results

### Gal-1 is down-regulated in the induced sputum of asthmatic patients and PDGF-BB-stimulated ASMCs

**Figure 1:**
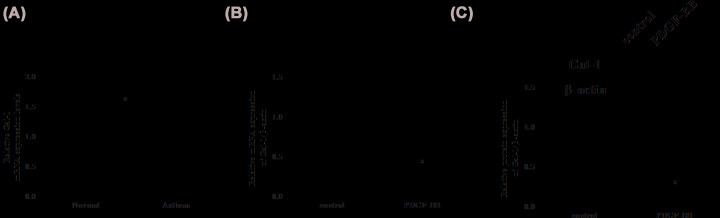
Gal-1 expression is decreased in the induced sputum of asthmatic patients and PDGF-BB-stimulated ASMCs Comparison of Gal-1 levels in the induced sputum from asthma patients (*n* = 24) and healthy control subjects (*n* = 18). (**A**) The mRNA expression levels of Gal-1 were detected using qRT-PCR. *P<0.05 vs. healthy control subjects. ASMCs were stimulated with PDGF-BB (10 ng/ml) for 24 h. (**B,C**) The expression levels of Gal-1 were detected using qRT-PCR and Western blot. *P<0.05 vs. control ASMCs.

We first evaluated the mRNA levels of Gal-1 in the induced sputum using quantitative RT-PCR (qRT-PCR). The results showed that compared with the control group, Gal-1 mRNA levels were lower in the induced sputum of asthma patients (Figure 1A). In addition, the expressions of Gal-1 in cultured ASMCs were also detected by qRT-PCR and Western blot. As indicated in Figure 1B,C, the expressions of Gal-1 at both mRNA and protein levels were significantly decreased by PDGF-BB in ASMCs.

### Overexpression of Gal-1 inhibits PDGF-BB-induced ASMCs proliferation and migration

**Figure 2:**
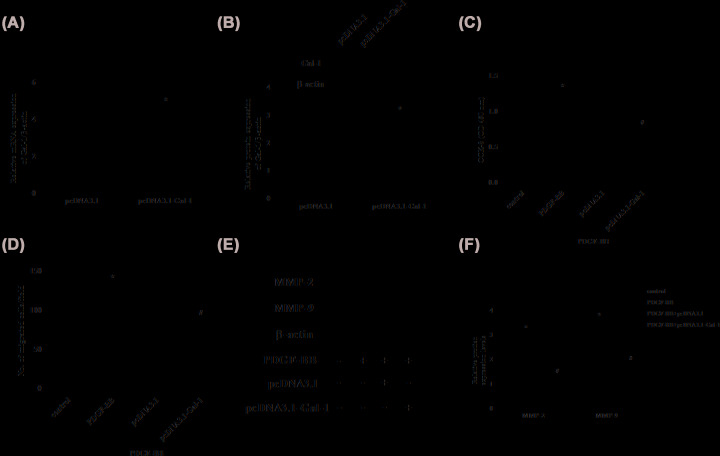
Effect of Gal-1 overexpression on PDGF-BB-induced proliferation and migration of ASMCs (**A,B**) Successful establishment of Gal-1-overexpressing ASMCs. ASMCs were transfected with pcDNA3.1-Gal-1 vector or pcDNA3.1 vector. After 48 h post transfection, cells were collected for qRT-PCR and Western blot analysis. (**C,D**) CCK-8 and transwell assays were carried out to measure the cell proliferation and migration of ASMCs after incubated with or without PDGF-BB (10 ng/ml) for 24 h. (**E**) Western blot analysis was performed to evaluate the expressions of MMP-2 and MMP-9. (**F**) Quantification analysis of MMP-2 and MMP-9. *P<0.05 vs. ASMCs transfected with pcDNA3.1 vector; ^#^*P*<0.05 vs. PDGF-BB-treated ASMCs. For each replicate (*n* = 4), the experiment was performed in triplicate.

To further explore the function of Gal-1, ASMCs well transfected with pcDNA3.1-Gal-1 vector to overexpress Gal-1. As shown in Figure 2A,B, Gal-1 expression in ASMCs was markedly increased by pcDNA3.1-Gal-1, as compared with pcDNA3.1-transfected ASMCs. CCK-8 assay proved that overexpression of Gal-1 significantly suppressed ASMCs proliferation in response to PDGF-BB (Figure 2C). Transwell assay showed that PDGF-BB-induced ASMCs migration was greatly reduced by Gal-1 overexpression (Figure 2D). Additionally, the PDGF-BB-induced expressions of MMP-2 and MMP-9 were also attenuated by Gal-1 (Figure 2E,F).

### Knockdown of Gal-1 enhances PDGF-BB-induced ASMCs proliferation and migration

**Figure 3:**
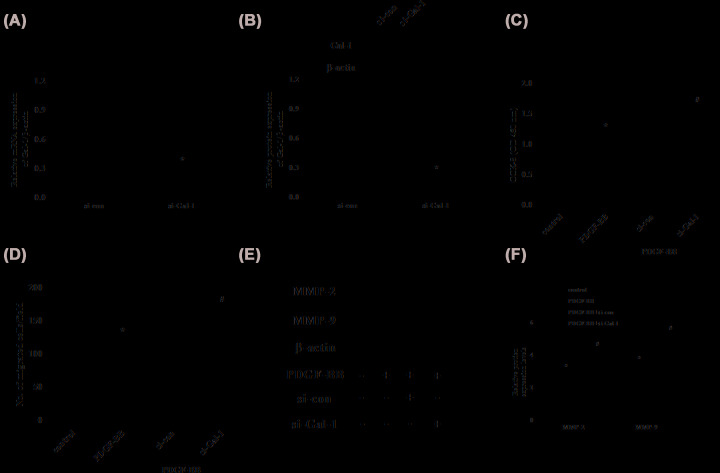
Effect of Gal-1 knockdown on PDGF-BB-induced proliferation and migration of ASMCs (**A,B**) Successful establishment of Gal-1-knockdown ASMCs. ASMCs were transfected with si-Gal-1 or si-control. After 48 h post transfection, ASMCs were collected for qRT-PCR and Western blot analysis. (**C**) ASMCs with different transfections were incubated with or without PDGF-BB (10 ng/ml) for 24 h, CCK-8 assay was carried out to measure the cell proliferation. (**D,E**) Cell migration was evaluated by transwell assay and detecting the expressions of MMP-2 and MMP-9 using Western blot analysis. (**F**) Quantification analysis of MMP-2 and MMP-9. *P<0.05 vs. ASMCs transfected with si-control; ^#^*P*<0.05 vs. PDGF-BB-treated ASMCs. For each replicate (*n* = 5), the experiment was performed in triplicate.

Besides, ASMCs were transfected with si-Gal-1 to knock down Gal-1. Gal-1 expression was dramatically reduced by si-Gal-1 in ASMCs when compared with si-control-transfected ASMCs (Figure 3A,B). As shown in Figure 3C,D, knockdown of Gal-1 elevated the proliferative and migrative abilities in PDGF-BB-induced ASMCs. In addition, the PDGF-BB-caused increases in expressions of MMP-2 and MMP-9 were enhanced by Gal-1 silencing (Figure 3E,F).

### Overexpression of Gal-1 enhances contractile phenotype markers in PDGF-BB-induced ASMCs

**Figure 4:**
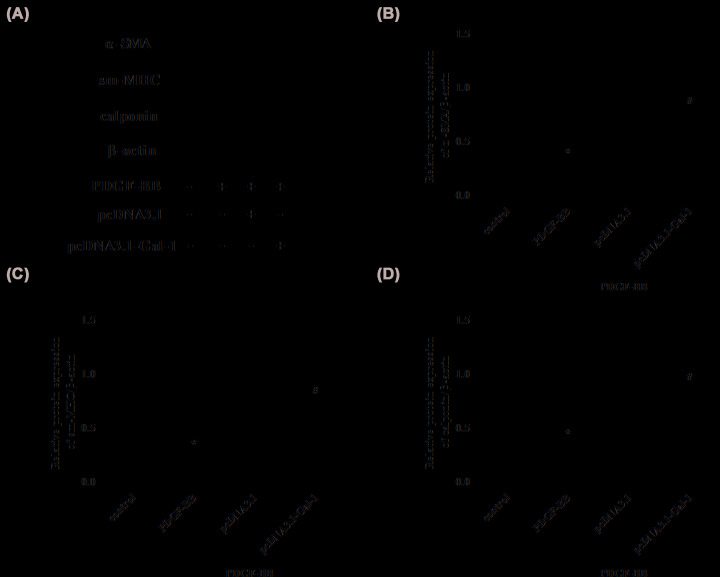
Effect of Gal-1 overexpression on PDGF-BB-induced phenotype switching of ASMCs ASMCs were transfected with pcDNA3.1-Gal-1 vector or pcDNA3.1 vector, and then incubated with or without PDGF-BB (10 ng/ml) for 24 h. (**A**) The expressions of contractile phenotype markers including α-SMA, sm-MHC, and calponin were measured by Western blot. (**B–D**) Quantification analysis of α-SMA, sm-MHC, and calponin. *P<0.05 vs. ASMCs transfected with pcDNA3.1 vector; ^#^*P*<0.05 vs. PDGF-BB-treated ASMCs. For each replicate (*n* = 4), the experiment was performed in triplicate.

To investigate the role of Gal-1 in phenotypic plasticity of ASMCs in response to PDGF-BB, the expressions of α-SMA along with sm-MHC, and calponin were measured by Western blot. The results indicated that PDGF-BB stimulation caused significant decrease in expressions of α-SMA, sm-MHC, and calponin in ASMCs. However, overexpression of Gal-1 elevated the expressions of contractile phenotype markers in PDGF-BB-induced ASMCs (Figure 4).

### Knockdown of Gal-1 aggravates PDGF-BB-induced phenotype switching of ASMCs

**Figure 5:**
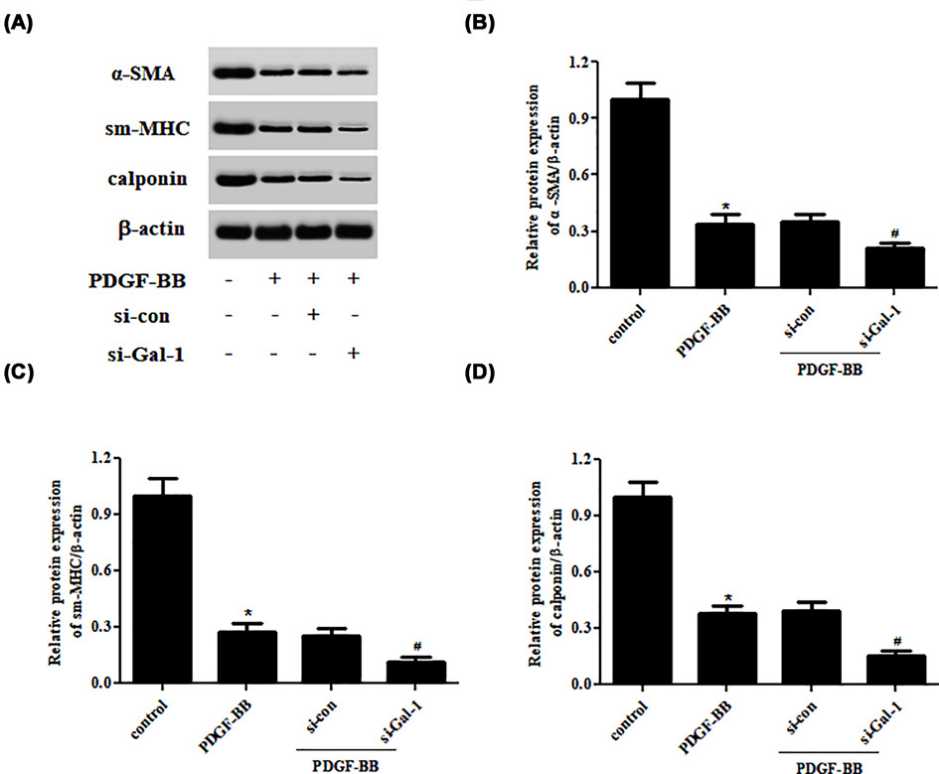
Effect of Gal-1 knockdown on PDGF-BB-induced phenotype switching of ASMCs ASMCs were transfected with si-Gal-1 or si-control, followed by an incubation with or without PDGF-BB (10 ng/ml) for 24 h. (**A**) The expressions of α-SMA, sm-MHC, and calponin were measured by Western blot. (**B–D**) Quantification analysis of α-SMA, sm-MHC, and calponin. *P<0.05 vs. ASMCs transfected with si-control; ^#^*P*<0.05 vs. PDGF-BB-treated ASMCs. For each replicate (*n* = 5), the experiment was performed in triplicate.

In contrast with the effects of Gal-1 overexpression on the expressions of contractile phenotype markers, knockdown of Gal-1 resulted in remarkable reductions in expressions of α-SMA, sm-MHC, and calponin in PDGF-BB-induced ASMCs (Figure 5).

### Overexpression of Gal-1 inhibits PI3K/Akt activation in PDGF-BB-stimulated ASMCs

**Figure 6:**
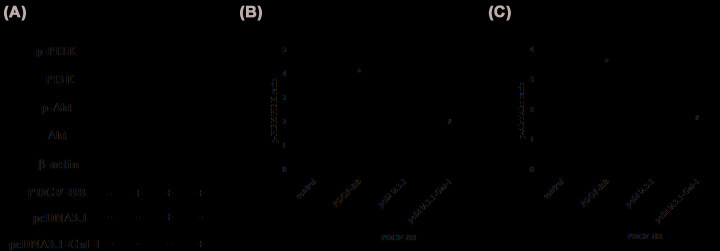
Effect of Gal-1 overexpression on PDGF-BB-induced PI3K/Akt activation in ASMCs (**A**) After transfection with pcDNA3.1-Gal-1 vector or pcDNA3.1 vector, and the following stimulation with or without PDGF-BB (10 ng/ml) for 24 h, the expressions of PI3K, p-PI3K, Akt, and p-Akt in ASMCs were detected by Western blot. (**B**) The ratio of p-PI3K/PI3K. (**C**) The ratio of p-Akt/Akt. *P<0.05 vs. ASMCs transfected with pcDNA3.1 vector; ^#^*P*<0.05 vs. PDGF-BB-treated ASMCs. For each replicate (*n* = 4), the experiment was performed in triplicate.

Next, we assessed the effect of Gal-1 on the activation of PI3K/Akt signaling pathway through detection the expressions of PI3K, p-PI3K, Akt, and p-Akt. Western blot showed that the expressions of p-PI3K and p-Akt were markedly increased by PDGF-BB in ASMCs compared with the control group. However, the increased p-PI3K and p-Akt expressions were prevented by overexpression of Gal-1 (Figure 6).

### PI3K activator reversed the effects of Gal-1 on ASMCs proliferation, migration, and phenotype switching

**Figure 7:**
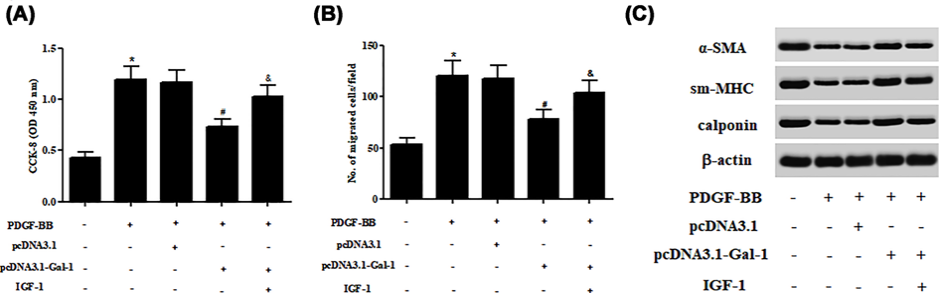
Effect of IGF-1 on proliferation, migration, and phenotype switching in Gal-1-overexpressing ASMCs ASMCs were treated with IGF-1 (5 μM) to activate PI3K/Akt signaling pathway in Gal-1 overexpressed ASMCs. (**A,B**) Comparison of cell proliferation and migration using CCK-8 and transwell assays. (**C**) Comparison of α-SMA, sm-MHC, and calponin expressions using Western blot. *P<0.05 vs. control group; ^#^*P*<0.05 vs. PDGF-BB group; ^&^*P*<0.05 vs. PDGF-BB+pcDNA3.1-Gal-1 group. For each replicate (*n* = 4), the experiment was performed in triplicate.

Subsequently, ASMCs were treated with IGF-1 to activate the PI3K/Akt signaling pathway. We observed that the inhibitory effects of Gal-1 on ASMCs proliferation and migration were abolished by IGF-1 treatment (Figure 7A,B). In addition, IGF-1 treatment caused significant decreases in the expressions of α-SMA, sm-MHC, and calponin in Gal-1 overexpressed ASMCs (Figure 7C).

## Discussion

Galectins are members of animal lectin protein family, with an extensive family of conserved glycan binding proteins [17]. Galectins have multiple roles in modulating responses in innate and adaptive immunity, cancer and chronic inflammation, and act as molecular targets for therapeutic intervention [18–20]. In addition to these roles, some galectins are found to participate in modulating allergic inflammatory response in the airways [18,21]. Gal-1 is an important member of galectins family widely distributed in many types of cells and exerts broad roles intracellularly or extracellularly. Increasing evidence has indicated that Gal-1 is involved in the pathogenesis of asthma [15]. Gal-1 shows a reduced expression on macrophages from sputum samples of asthma patients compared with cells from healthy donors. *In vitro* immunoassays prove that Gal-1 is able to induce the production of IL-10 by peripheral blood mononuclear cells from healthy donors [22]. Ge et al. [14] reported that the expression of Gal-1 is induced in allergic lungs. Gal-1 limits eosinophil recruitment to allergic airways and suppresses airway inflammation via suppressing cell migration and promoting eosinophil apoptosis. In the current study, we found that Gal-1 was markedly down-regulated in the induced sputum of asthma patients.

Previous findings suggest that Gal-1 participates in the pathogenesis of asthma through regulation of inflammatory response. In addition to the inflammation, ASMCs hyperplasia has been regarded as the main mechanism of asthma. It has been reported that Gal-1 restricts vascular smooth muscle cells (VSMCs) migration, which plays a key role in the development of intimal hyperplasia and atherosclerosis [23]. Thus, we aimed to investigate the effect of Gal-1 on ASMCs. PDGF-BB is one of the dimeric isoforms that has a prominent role in promoting the changes of ASMCs from a contractile phenotype to a pro-remodeling phenotype with proliferative, migrative and synthetic capacities [24–26]. Therefore, we used PDGF-BB to induce the phenotypic change of ASMCs. Our results showed that Gal-1 overexpression inhibits PDGF-BB-induced ASMCs proliferation and migration, while Gal-1 knockdown exhibits opposite effects of Gal-1 overexpression. In addition, the reduced expressions of contractile phenotype markers including α-SMA, sm-MHC, and calponin were elevated by Gal-1 overexpression, but decreased by Gal-1 knockdown in ASMCs. The results indicate that Gal-1 prevented PDGF-BB-induced phenotypic switching of ASMCs.

PI3K/Akt pathway is an important intracellular signaling pathway that has been found to be abnormal activated in various kinds of diseases including asthma [27–29]. Emerging evidences suggest that inhibition of the PI3K/Akt signaling pathway has positive effects on suppression of ASMCs phenotype modulation, implying that targeting PI3K/Akt pathway may be a novel therapy to asthma. Xie et al. [30] demonstrated that esculetin regulates the PDGF-induced phenotype switching of ASMCs through inhibition of PI3K/Akt pathway. MicroRNA-200a suppresses ASMCs proliferation and airway remodeling in asthmatic mice via inhibiting the activation of the PI3K/Akt signaling pathway [31]. The present study showed that overexpression of Gal-1 inhibits PI3K/Akt activation in PDGF-BB-stimulated ASMCs. However, treatment with PI3K activator reversed the effects of Gal-1 on ASMCs proliferation, migration and phenotype switching. These findings suggested that Gal-1 exerted its suppression role in PDGF-BB-stimulated ASMCs via inhibiting PI3K/Akt signaling pathway.

There existed several limitations in the present study. First, we only evaluated the effect of Gal-1 on PDGF-BB-stimulated ASMCs *in vitro*. An *in vivo* animal study will be considered in the following studies. Second, whether glucocorticoids affect the effect of Gal-1 on phenotype switching of ASMCs will require further experiments.

Taken together, our study revealed that Gal-1 exerted an inhibitory role in PDGF-BB-stimulated proliferation, migration and phenotype switching of ASMCs. This is likely through regulating the PI3K/Akt signaling pathway. Our work strongly suggests that Gal-1 might be a promising target for the treatment of asthma.

## Abbreviations

ASMC: airway smooth muscle cell
CCK-8: Cell Counting Kit-8
DMEM: Dulbecco’s modified Eagles medium
FBS: fetal bovine serum
Gal-1: galectin-1
PDGF-BB: platelet-derived growth factor BB
qRT-PCR: quantitative RT-PCR
SM-MHC: specific muscle myosin heavy chain
α-SMA: α-smooth muscle actin
